# Macroautophagy Abnormality in Essential Tremor

**DOI:** 10.1371/journal.pone.0053040

**Published:** 2012-12-27

**Authors:** Sheng-Han Kuo, Guomei Tang, Karen Ma, Rachel Babij, Etty Cortes, Jean-Paul G. Vonsattel, Phyllis L. Faust, David Sulzer, Elan D. Louis

**Affiliations:** 1 Department of Neurology, College of Physicians and Surgeons, Columbia University, New York, New York, United States of America; 2 GH Sergievsky Center, College of Physicians and Surgeons, Columbia University, New York, New York, United States of America; 3 Taub Institute for Research on Alzheimer's Disease and the Aging Brain, College of Physicians and Surgeons, Columbia University, New York, New York, United States of America; 4 Department of Pathology and Cell Biology, Columbia University Medical Center and the New York Presbyterian Hospital, New York, New York, United States of America; 5 Department of Epidemiology, Mailman School of Public Health, Columbia University, New York, New York, United States of America; Kaohsiung Chang Gung Memorial Hospital, Taiwan

## Abstract

Macroautophagy is a cellular mechanism for the clearance of protein aggregates and damaged organelles. Impaired macroautophagy has been observed in neurodegenerative disorders. We investigated the macroautophagy pathway in essential tremor (ET) cases compared to age-matched controls. We analyzed microtubule-associated protein light chain 3-II (LC3-II), S6K, phosphorylated S6K, beclin-1, and mitochondrial membrane proteins levels by Western blot in the post-mortem cerebellum of 10 ET cases and 11 controls. We also performed immunohistochemistry in 12 ET cases and 13 controls to quantify LC3 clustering in Purkinje cells (PCs). LC3-II protein levels were significantly lower in ET cases vs. controls on Western blot (0.84±0.14 vs. 1.00±0.14, p = 0.02), and LC3-II clustering in PCs by immunohistochemistry was significantly lower in ET cases vs. controls (2.03±3.45 vs. 8.80±9.81, p = 0.03). In ET cases, disease duration was inversely correlated with LC3-II protein level (r = −0.64, p = 0.046). We found that mitochondrial membrane proteins were accumulated in ET (TIM23: 1.36±0.11 in ET cases vs. 1.00±0.08 in controls, p = 0.02; TOMM20: 1.63±0.87 in ET cases vs. 1.00±0.14 in controls, p = 0.03). Beclin-1, which is involved in macroautophagy, was strikingly deficient in ET (0.42±0.13 vs. 1.00±0.35, p<0.001). Decreased macroautophagy was observed in the ET cerebellum, and this could be due to a decrease in beclin-1 levels, which subsequently lead to mitochondrial accumulation as a result of autophagic failure. This provides a possible means by which perturbed macroautophagy could contribute to PC pathology in ET.

## Introduction

Essential tremor (ET) is among the most prevalent movement disorders [1]. In postmortem studies, degenerative changes in the cerebellum, including an increase in the number of Purkinje cell (PC) axonal torpedoes and PC loss have been reported [2], [3]. Other pathological features have also been reported in ET, including an increase in the numbers of heterotopic PCs, an increased density of the basket cell axonal plexus surrounding PCs, and Bergmann gliosis [4]–[6]. In contrast, granule cells and parallel fibers seem to be relatively preserved in ET [7]. Whether ET is a neurodegenerative disease is under active discussion [8].

Since PC loss has been reported in ET cerebellum, we explored potential mechanisms of such PC loss. The main mechanisms of PC death are apoptosis, autophagy, and necrosis [9]. Autophagy is of particular interest since many neurodegenerative diseases are characterized by autophagic alterations that are linked to proteinacious accumulations as well as neuronal death [10]. One of the autophagic pathways, macroautophagy, is a cellular degradative process in which organelles such as mitochondria and aggregated proteins are engulfed by double-membraned vacuoles (AVs) that are subsequently targeted for degradation in lysosomes. A direct link between autophagy and neurodegeneration has been established by loss of basal autophagy in mouse brains through conditional knockout of key autophagy genes, *Atg5* and *Atg7*; this results in neurodegenerative phenotypes with accumulation of ubiquitinated aggregates and neuronal loss [11], [12]. Mutations or overexpression in neurodegenerative disease genes, including presenilin [13], huntingtin (Htt) [14], α-synulcien [15], [16], parkin, and PINK1 [17], have been reported to inhibit macroautophagy. These studies highlight the importance of autophagy in neuronal homeostasis and survival. In this study, we investigated whether changes in autophagy occur in the cerebellum of ET cases compared to that of age-matched controls.

## Methods

### Ethics statement

All the brain donors signed the informed consent approved by Columbia institutional review board to donate their brains for scientific research. All samples were de-identified and analyzed anonymously.

### Brain Repository and Study Subjects

The study was conducted at the Essential Tremor Centralized Brain Repository (ETCBR) [18]. Postmortem cerebellar tissue was obtained from ET cases and age-matched controls. All brains received a comprehensive neuropathological diagnostic assessment as previously described [19].

The clinical diagnosis of ET, initially assigned by treating neurologists, was confirmed by ETCBR study neurologists using a detailed, videotaped, in-person neurological assessment that was followed by application of ETCBR diagnostic criteria [18], which required the presence of moderate or greater amplitude kinetic arm tremor that was not attributable to Parkinson disease (PD) or dystonic tremor. Control brains were from individuals followed at the Alzheimer Disease Research Center or the Washington Heights Inwood Columbia Aging Project. They were followed prospectively with serial neurological examinations and were clinically free of Alzheimer Disease (AD), ET, PD, dementia with Lewy bodies (DLB), or progressive supranuclear palsy, and their brains were without diagnostic abnormalities on standardized neuropathological evaluation. The number of ET cases and controls in each experiment are shown in Table 1.

**Table 1:** Clinical and pathological features of ET cases and controls.

	Cerebellar cortex				Occipital cortex	
	Western Blot Analysis		Immunohistochemistry		Western Blot Analysis	
	ET	Controls	ET	Controls	ET	Controls
N	10	11	12	13	7	9
Age at death (years)	85.7±6.1	84.5±6.4	86.5±6.4	83.0±7.6	84.3±8.8	84.8±6.3
Female Gender	5 (50.0%)	6 (54.5%)	8 (75%)	7 (58.3%)	3 (42.9%)	5 (55.6%)
Brain Weight (grams)	1211±126	1174±145	1187±123	1231±140	1207±140	1175±157
Postmortem Interval (hours)	3.1±2.3	4.7±2.3	2.6±1.8	8.9±10.5^A^	4.4±3.8	4.1±1.7
Braak AD Stage	2.0±1.2	2.0±1.1	2.5±1.2	1.7±1.2	1.6±1.0	2.0±1.1
CERAD Plaque Score						
0	5 (50.0%)	5 (45.5%)	7 (58.3%)	7 (53.8%)	4 (57.1%)	5 (55.6%)
A	3 (30.0%)	3 (27.3%)	3 (25.0%)	4 (30.8%)	1 (14.2%)	1 (11.1%)
B	2 (20.0%)	3 (27.3%)	2 (16.7%)	2 (15.3%)	2 (28.6%)	3 (33.3%)
C	0 (0.0%)	0 (0.0%)	0 (8.3%)	0 (0.0%)	0 (0.0%)	0 (0.0%)
Purkinje cell counts	7.3±2.6	8.5±2.2	6.2±0.8	9.0±2.6	7.5±2.6	10.2±3.4
Axonal Torpedoes*	23.9±24.8	4.4±2.2	29.8±28.1	3.6±2.1	14.9±1.4	2.6±1.4

* p<0.05.

A Two controls had PMI >15 hours. Median PMI in controls = 5.3 hours.

### Western Blot

Frozen brain samples in standardized vials were solubilized in RIPA buffer (Sigma) with protease and phosphatase inhibitors, and were sonicated and subsequently centrifuged at 16870 g for 30 minutes. The supernatant was used for analysis. An equal amount of protein from each brain homogenate was separated on a NuPAGE 4–12% Gel (Invitrogen) and transferred to a PVDF membrane (Millipore). We used the following antibodies: β-actin (1∶1000, Sigma), LC3 (Novus Biologicals 1384 1∶1000), and calbindin (1∶1000, Sigma). The LC3 antibody has been extensively used to study AVs in postmortem human brains [15], [20]. We used LC3-II specific antibody (Novus Biologicals 19167, 1∶000) to confirm the specificity. The secondary antibodies were conjugated with horseradish peroxidase (Thermo scientific). We used ECL (Millipore) to detect the signals, which were quantified in Image J (National Institutes of Health). Each experiment was repeated three times to obtain an average value for each sample.

### Tissue Processing and Immunohistochemistry

A standard 3×20×25 mm parasagittal neocerebellar block was harvested from the same region of each brain. Paraffin sections (7 µm thick) were stained with Luxol Fast Blue Hematoxylin and Eosin (LH&E) as described previously [2], [3]. Axonal torpedoes were also quantified in the entire LH&E-stained section [3].

Antigen retrieval of cerebellar sections was performed in Trilogy (Cell Marque) for 40 minutes, 100°C and sections were immunostained using anti-LC3 antibody (Novus Biologicals 1384, 1∶100) at 4°C for 48 hours followed by Alexa 488 conjugated secondary antibody (Invitrogen). Calbindin staining was performed with monoclonal mouse antibody (Abcam, 1∶100) and Alexa 594 conjugated secondary antibody (Invitrogen) In addition, we also used the secondary antibody conjugated with horseradish peroxidase with 3,3′-diaminobenzidine (DAB). We used another LC3-II specific antibody (Abcam ab58610, 1∶100), which also showed a similar staining pattern. Immunohistochemistry with the omission of primary antibody was used as a negative control, which did not show significant staining.

The central folia of each cerebellar section were identified and five PCs in each slide were randomly chosen within the central folia. Images were obtained by confocal microscopy (Leica, 63X) with Ar 488/HeNEL 543 laser. A trained physician (SHK), who was blinded to clinical and diagnostic data, obtained all images with the same acquisition settings. Images were analyzed by Image J. The AVs (LC3 puncta) were quantified as previously described [21]. Briefly, PCs were identified by their morphology, their distinct localization between the molecular and granule cell layers, and their positive staining of calbindin. We first compared the Z-stack composite image for the whole thickness of the section and a single optical slide, and found their LC3 staining patterns were similar. Therefore, we elected to use a single optical slide for AV quantification. Images were analyzed by Image J (National Institutes of Health, Bestheda). The AVs were identified as the LC3 positive structures within PC cell bodies. We first randomly selected 5 background values from the molecular layer and chose the 40 points above the average background value as the threshold for AV quantification. All the pixels above the threshold and within PC cell bodies were quantified. The usual size of AVs is 0.1–10 µm in diameter, but many AVs in PCs are either fused with or close to each other. Therefore, it is difficult to quantify the actual numbers of AVs. Instead, we summed the pixels above the threshold value and divided by the cell body area, excluding the nucleus, to obtain the percentage of cell body area occupied by AVs. We also used a second analytic method, in which we used a set threshold value for all the images, and calculated the percentage of cell body area occupied by AVs; this analysis showed similar results.

### Data Analyses

Analyses were performed in SPSS (version 18.0) and GraphPad Prism (version 5.0). Demographic and clinical characteristics of ET cases and controls were compared using Student's t tests and chi square tests. The mean LC3-II protein levels, mitochondrial membrane protein levels, and beclin-1 levels (Western blot) and the percentage of cell bodies occupied by AVs (immunohistochemistry) were normally distributed; hence, parametric tests (Student's t test, Pearson's correlation coefficient [r]) were used when assessing these variables. Based on the presence in our sample of a clear bimodal distribution in disease duration among ET cases (≤40 years vs. >40 years), study subjects were stratified into 3 diagnosis-duration groups: controls, ET cases with shorter duration disease, and ET cases with longer duration disease. In linear regression models, we examined the association between LC3-II protein level or the percentage of cell bodies occupied by AVs (dependent variables in different models) and the diagnosis-duration group (controls, ET of shorter duration, ET of longer duration).

## Results

**Figure 1:**
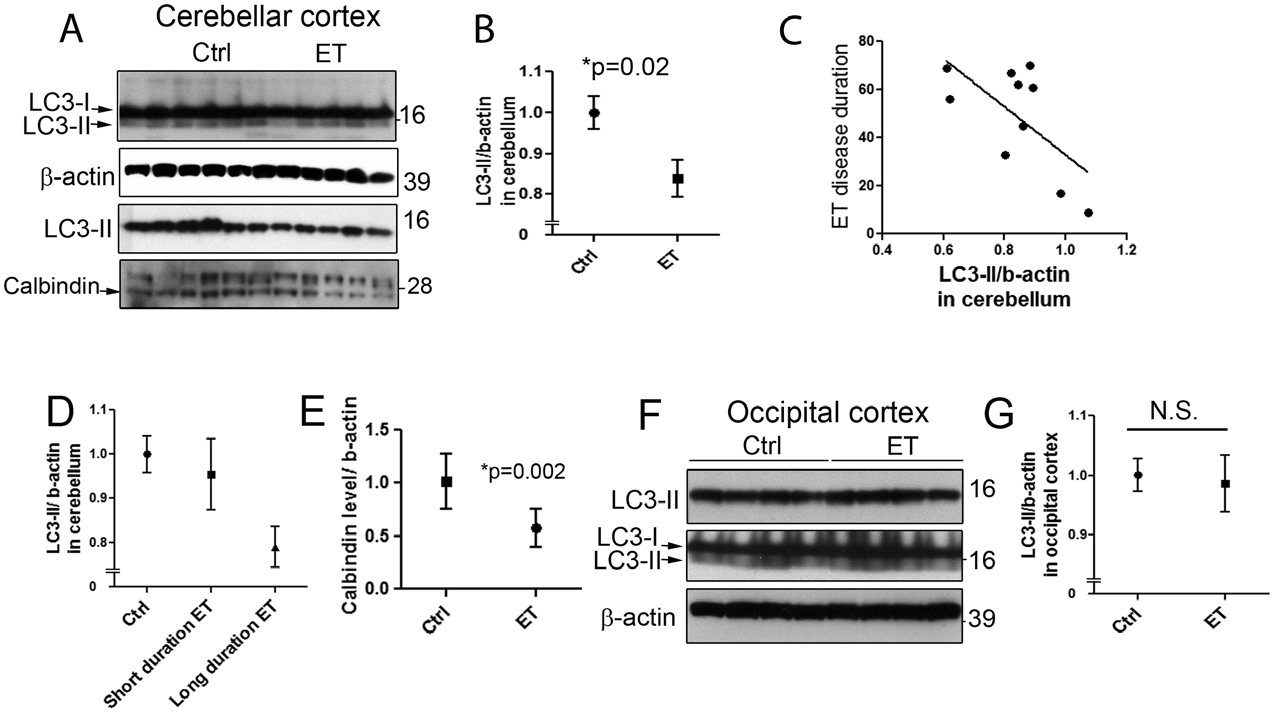
Decreased LC3-II levels in the cerebellum of essential tremor (ET) cases and controls. LC3-II and β-actin levels in cerebellar homogenates were determined by Western blot in 10 ET cases and 11 controls. Two LC3 antibodies were used, LC3 antibody, Novus Biologicals 1384 (top panel), and LC3-II specific antibody Novus Biologicals 19167 (third panel) and the representative bands were shown (A). LC3-II levels (mean ± SD) were significantly lower in ET cases vs. controls (B). In a linear regression model, ET disease duration inversely correlated with LC3-II level (C).When dividing our sample into three categories, ET cases with the longest disease duration had the most diminished LC3-II levels, followed by ET cases with shorter duration disease and then controls (D). ET cases displayed lower levels of calbindin than age-matched controls, consistent with PC cell loss (A, E). We also determined the LC3-II and β-actin levels in the occipital cortex in 7 ET cases and 9 controls and the representative blots were shown (F). ET cases and controls exhibited similar LC3-II levels in the occipital cortex (mean ± SD) (G).

Cerebellar tissue was available for Western blot analysis on 10 ET cases and 11 age-matched controls who were similar with respect to age, gender, brain weight and other variables of interest (Table 1). The mean LC3-II protein level determined by Western blot with LC3 antibody was lower in ET cases than controls (0.84±0.14 vs. 1.00±0.14, p = 0.02)(Figure 1A, B). We used another LC3-II specific antibody (see Methods) and found the similar case-control differences (p = 0.01) and there was a high correlation between the LC3-II levels detected by two antibodies (r = 0.52, p = 0.01)

Among ET cases, disease duration was inversely correlated with LC3-II protein level (r = −0.64, p = 0.046) (Figure 1C). The mean disease duration was 49.9±22.2 years (range = 9–70 years). Based on the presence of a clear bimodal distribution in disease duration among ET cases in our sample (≤40 years vs. >40 years), study subjects were stratified into 3 diagnosis-duration groups: controls, ET cases with shorter duration disease (n = 3, mean = 19.7±12.2 years, range = 9–33 years), and ET cases with longer duration disease (n = 7, mean = 62.9±6.0 years, range = 55–70 years). The respective LC3-II protein levels were: 1.00±0.14, 0.95±0.14, and 0.79±0.12, and in a linear regression model, LC3-II protein level declined by diagnosis-duration group (r^2^ = 0.37, p = 0.004) (Figure 1D). We also investigated calbindin level, a protein specifically expressed by PCs in the cerebellum, and found that ET cases had a lower level of calbindin than controls (0.58±0.18 vs. 1.01±0.26, p<0.01) (Figure 1E), consistent with our previous findings that ET cases had a lower number of PCs [3].

We then investigated whether decrease in LC3-II was specific to the ET cerebellum. We determined LC3-II protein level in the occipital cortex in 7 ET cases and 9 controls: ET cases had similar LC3-II level as controls (0.98±0.13 vs. 1.00±0.08) (Figure 1F, G).

**Figure 2:**
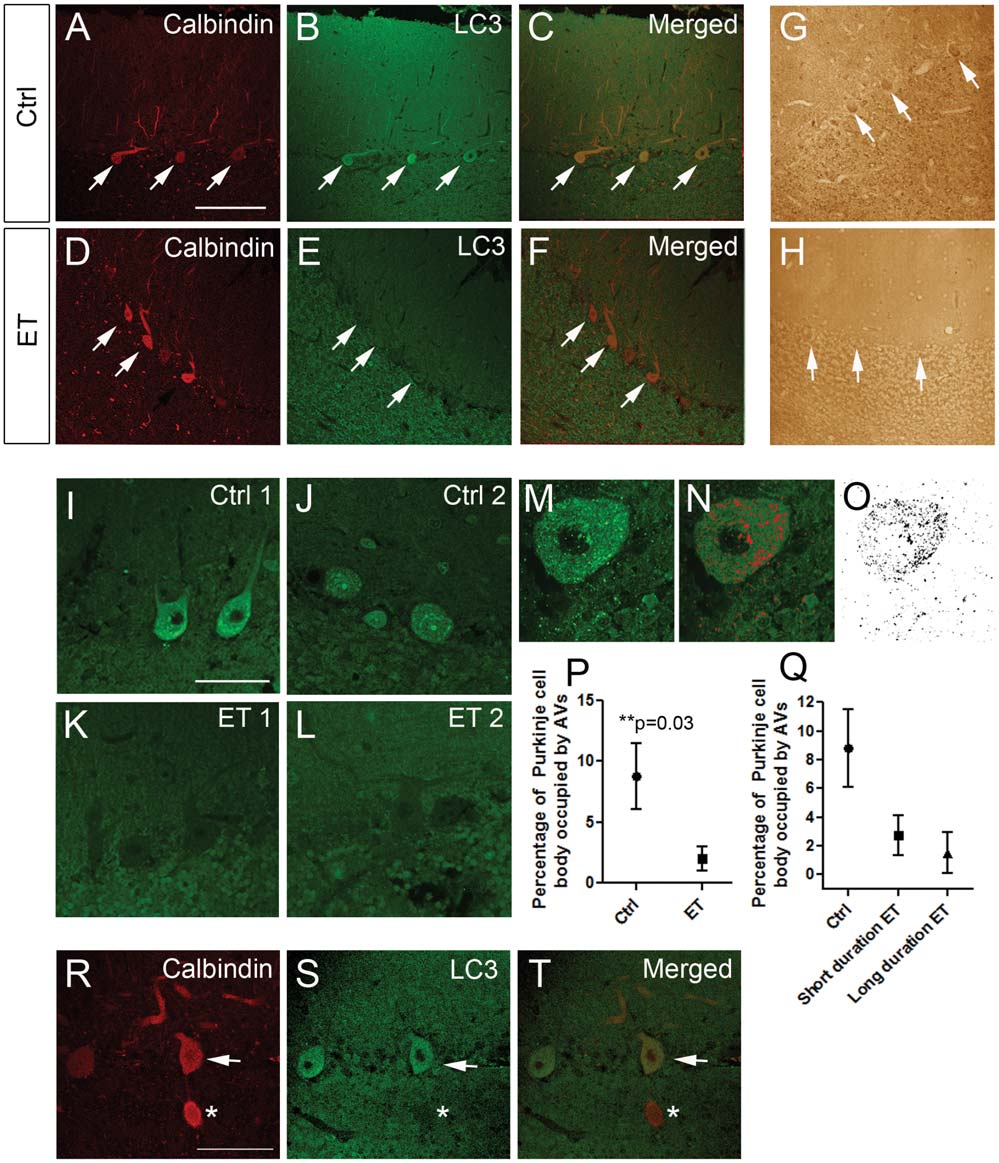
LC3-II immunohistochemistry in PCs was decreased in ET cases vs. controls. Cerebellar cortical sections from controls (A–C) and ET cases (D–F) were double immunolabelled with anti-calbindin and Alexa 594 (A, C, D, F, red), or with anti-LC3 and Alexa 488 (B, C, E, F, green) and imaged by confocal microscopy using the same acquisition parameters. LC3 signals are much stronger in PCs (white arrows) in control (B) than in ET case (E). We also labeled the cerebellar cortical sections with anti-LC3 antibody conjugated with avidin/biotin complex and horseradish peroxidase and stained with 3,3′-diaminobenzidine (DAB) (G, H, brown). PCs exhibited stronger immunolabelling with DAB in control (G) than ET case (H). Scale bar: 200 µm. Higher magnification confocal images of PCs stained with LC3 and Alexa 488 showed that controls (I, J) contained more LC3 puncta than ET cases (K, L). Scale bar: 50 µm. Using image J, we further analyzed the percentage of PC body occupied by AVs (M–O). The percentage of PC body occupied by AVs was significantly lower in ET cases than controls (P). We further divided our samples into three groups including controls, short duration ET group, and long duration ET group and compared the LC3-II clustering. LC3-II clustering was highest in the controls and lowest in the long duration ET group (Q). A cerebellar cortical section was stained with calbindin (R, red) and LC3 (S, green) in a case of ET. A PC body (arrow) and an axonal torpedo (asterisk) were identified by the positive calbindin staining (R). Axonal torpedo did not display any LC3 staining (S, T). Scale bar: 50 µm.

Cerebellar tissue was available for immunohistochemistry on 12 ET cases and 13 age-matched controls, who were similar with respect to age, gender, brain weight and other variables of interest (Table 1). These included 6 of the 10 ET cases and 8 of 11 controls used in the Western blot analysis. We labeled the cerebellar sections with anti-LC3 and anti-calbindin antibodies to assess the LC3 content in PCs. PCs were found to have a robust autophagic activity, reflected by LC3 clustering; therefore, we used LC3 staining to assessed the autophagic activity in PCs [22]. We found that PCs in ET cases exhibited lower LC3 staining (Figure 2A–H). We found that LC3 was present in punctate structures, which labels them as AVs: PCs in ET cases had strikingly fewer LC3 puncta than controls (Figure 2I–L). We quantified the fraction of PC bodies, excluding the nucleus, that was occupied by AVs (Figure 2M–O). The percentage of cell bodies occupied by AVs was more than 4-fold lower in ET cases than controls (2.03±3.45 vs. 8.80±9.81, p = 0.03)(Figure 2P). The results from Western blot analyses (i.e., LC3-II protein levels), were highly correlated with these immunolabel results (r = 0.78, p = 0.001).

Among the 12 ET cases with immunolabel results, the mean disease duration of the patients was 46.3±22.1 years (range = 17–80 years). Disease duration was not correlated with the fraction of cell bodies occupied by AVs (r = −0.12, p = 0.70), yet when study subjects were stratified into 3 diagnosis-duration groups (controls; ET cases with shorter duration disease [n = 4, mean = 19.8±3.4 years]; and ET cases with longer duration disease [n = 8, mean = 59.6±12.6 years]), the respective percentage of cell bodies occupied by AVs were: 8.80±9.81, 3.21±3.32, and 1.44±3.56, and in a linear regression model, the fraction of cell bodies occupied by AVs declined by diagnosis-duration group (r^2^ = 0.14, p = 0.035)(Figure 2Q). The number of torpedoes was not correlated with LC3-II protein levels on Western blot analysis (r = 0.04, p = 0.40) or with the percentage of cell bodies occupied by AVs on immunohistochemistry (r = 0.04, p = 0.33). We also found that axonal torpedoes in ET cases were also devoid of LC3 staining (Figure 2R–T).

We demonstrated a decreased LC3-II level in ET cerebellum and a decreased presence of AVs in PCs in ET. This could be due to either insufficient AV formation or increased AV clearance. To estimate effects on autophagic cargo in postmortem tissues [23], we examined mitochondria, which are degraded via macroautophagy. We reasoned that autophagic cargo accumulation would be consistent with insufficient AV formation in ET; in contrast, a decrease in autophagic cargo would be consistent with an accelerated AV clearance. Among the autophagic cargo, mitochondria mass has been most thoroughly studied in post-mortem human brain tissues. Indeed, autophagic cargo recognition failure leading to mitochondrial accumulation has been proposed to occur [14], and this has been confirmed in Hungtinton's disease (HD) post-mortem brain tissues [24].

**Figure 3:**
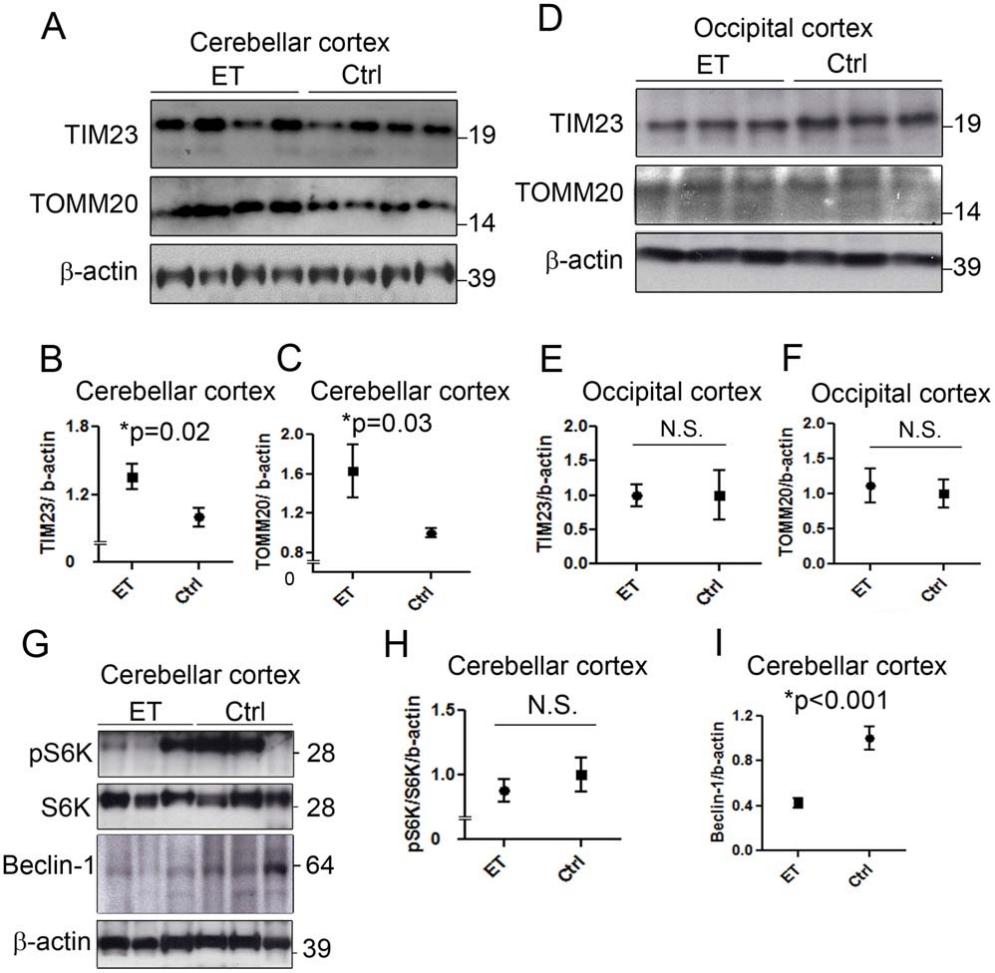
Mitochondrial accumulation and beclin-1 deficiency in ET cerebellum. Levels of mitochondrial membrane protein, TIM23 and TOMM20, in cerebellar cortex homogenates in 10 ET cases and 11 controls were determined by Western blot and the representative bands were shown (A). TIM23 and TOMM20 were normalized against β-actin to determine the protein levels. TIM23 and TOMM20 protein levels were significantly higher in the cerebellum of ET cases than controls (B, C). In contrast, TIM23 and TOMM20 protein levels were similar in the occipital cortex of 7 ET cases and 9 controls (D–F). S6K, pS6K, and beclin-1 levels in cerebellar cortex homogenates were determined by Western blot (G). pS6K levels were highly variable (G). pS6K and S6K ratio did not differ between ET cases and controls (H). Beclin-1 level was significantly lower in ET cases than controls (I).

We observed that the mitochondrial membrane proteins, translocase inner membrane 23 (TIM23), and translocase outer mitochondrial membrane 20 (TOMM20), were increased in the cerebellum in ET cases vs. controls (TIM23: 1.36±0.11 in ET cases vs. 1.00±0.08 in controls, p = 0.02; TOMM20: 1.63±0.87 in ET cases vs. 1.00±0.14 in controls, p = 0.03) (Figure 3A–C).This increase in mitochondrial mass suggests that the decrease in AVs observed in ET cerebellum may be due to impaired AV formation. In contrast, we found that similar mitochondrial protein levels were present in the occipital cortex of both ET cases and controls (TIM23: 1.00±0.16 in ET cases vs. 1.00±0.36 in controls; TOMM20: 1.11±0.25 in ET cases vs. 1.00±0.20 in controls) (Figure 3D–F).

We next investigated the two best characterized regulators of macroautophagy initiation: mammalian target of rapamycin (mTOR) and beclin-1. mTOR phosphorylates ULK1/2 and Atg13 complexes to inhibit autophagy, whereas beclin-1 is required for Vps34 and other protein complexes to induce autophagy [25]. Thus, mTOR serves to inhibit, and beclin-1, to promote macroautophagy. As we could not detect mTOR and phosphorylated mTOR on Western blot, like others [26], [27], possibly due to the large molecular weight of mTOR and the specificity of the antibodies against post-mortem human samples. We utilized the mTOR downstream effectors, phosphorylated S6K (pS6K) and S6K as reliable readouts for mTOR activity S6K is a ribosomal serine/threonine kinase and, upon phosphorylation by mTOR, S6K facilitates ribosomal biogenesis. ET cases had a similar pS6K/S6K ratio as controls (0.88±0.27 vs. 1.00±0.44, p = 0.47), suggesting that the differences in mTOR activity do not directly account for the decreased LC3-II in ET (Figure 3G, H).

In contrast, we found that beclin-1 level was decreased in ET cases vs. controls (0.42±0.13 vs. 1.00±0.35, p<0.0001)(Figure 3G, I). In a linear regression model, beclin-1 level was correlated with LC3-II level (r^2^ = 0.46, p<0.001), suggesting that beclin-1 could be an important rate-limiting molecule for AV formation in PCs and that beclin-1 deficiency could play a role in autophagic dysfunction in ET.

## Discussion

We observed lower LC3-II protein levels in the ET cerebellum and fewer AVs in the PCs in ET. These observations suggest that autophagic dysfunction could be a feature of ET. ET cases with the longest disease duration had the lowest LC3-II level and the most diminished AVs, followed by ET cases with shorter duration disease and then controls, indicating that the macroautophagic dysfunction might be related to ET disease duration. In addition, we showed that mitochondrial accumulation in ET, which is consistent with a reduced autophagic clearance of these organelles. The macroautophagy regulating protein, beclin-1, was moreover at very low levels in ET cerebellum, suggesting that beclin-1 deficiency might account for autophagic insufficiency in ET.

The early steps of AV formation involve the nucleation of double membranous structures followed by LC3-II recruitment; both mTOR and beclin-1 are important regulators in these autophagy initiation steps. Subsequent steps involve AV targeting to lysosomes and AV clearance. Inhibition of the early steps of macroautophagy can decrease AV formation whereas inhibition of later steps can lead to increased AV accumulation. Thus, inhibition of autophagy can result in either decreased or increased AVs.

In many neurodegenerative disorders, including AD, PD, HD, and DLB [14], [15], [20], [28]–[30], AV accumulation is evident in post-mortem brain tissue [14], [29]. This could result from impaired clearance of AVs due to the direct interference of autophagy by β-amyloid or Htt [13], [14]. In marked contrast with these other disorders, we observed that ET cases exhibited decreased levels of AVs when compared with controls. We further found a decreased beclin-1 level in ET cerebellum, consistent with an early step of autophagic failure, which further sets ET apart from other neurodegenerative disorders such as AD, PD, HD, or DLB [15], [20], [28], [30].

By forming the core complex required for AV formation, beclin-1 is an important player in the induction of macroautophagy [25]. Deficiency in beclin-1 has been observed in post-mortem AD brains and spinocerebellar ataxia type 3 (SCA3) patients' fibroblasts [31], [32]. Furthermore, beclin-1 is recruited to Htt inclusions in HD mouse model brains and in the striatum in HD patients, in which the reduced availability of beclin-1 might result in cell death [33]. Lentiviral delivery of beclin-1 in AD, PD, and SCA3 mouse models results in removal of amyloid β (Aβ), α-synuclein, and ataxin-3 aggregates, respectively [16], [31], [32]. Finally, beclin-1 plays an important role in PC degeneration, as mutated GluRδ in *lurcher* mice binds to nPIST and recruits beclin-1, which triggers autophagic cell death in PCs [34]. Together, these studies suggest that beclin-1 is an important regulator in neurodegenerative diseases.

The early steps of macroautophagy also involve two important cellular machinery proteins, Atg5 and Atg7 [35], which are required for AV formation and LC3-II clustering [36], [37]. Interestingly, *Atg5* or *Atg7* PC-specific deficient mice, which lack macroautophagy in PCs, showed age-dependent PC loss and PC axonal terminal swelling [36], [37]. In contrast with other mutant mice with PC degeneration, these mice only exhibit moderate PC loss and mild ataxia. Therefore, autophagic activities are essential for PC survival and PC axonal integrity, and autophagic failure could contribute to the PC pathology in ET. We note that in *Atg5* or *Atg7* PC-specific knockout mice, PC axonal swellings occurred at the distal end of the axons (at the level of the dentate nucleus) whereas most of the PC axonal torpedoes in ET have been observed in the proximal axons, and so the relationship between these features is not yet clear [3], [36], [37]. Nonetheless, autophagic activities are still important in maintaining PC axonal integrity.

Axonal torpedoes in ET represent the intracellular accumulation of neurofilament proteins, and we expected to find LC3 staining since AVs have been found to surround Lewy bodies and Htt aggregates [38], [39]. To our surprise, axonal torpedoes were devoid of LC3 immunolabel, which is consistent with the lack of double membranous structures surrounding organelles in axonal torpedoes [40].

One possible limitation of this study is that PCs constitute only a small percentage of cells in the cerebellar cortex and the results from Western blot analysis also reflect other cell types, such as granule cells, suggesting that other cell types might also have autophagy dysfunctions. Other limitations include the lack of direct visualization of AVs by electron microscopy (EM) [23]. It is however difficult to assess AVs in the postmortem human tissues due to the disruption of membranous structures and morphology of AVs. Boland et al were able to directly visualize AVs under EM from direct biopsy from a live patient's brain [30]. However, the current ET pathology materials do not allow us to conduct such a study, and therefore, we studied LC3-II levels by Western blot and LC3 clustering in immunohistochemistry.

The present data do not indicate if the apparent macroautophagy failure could be a secondary event to the primary cause of ET pathology and we do not rule out the possibility that beclin-1 deficiency could be due to upstream molecular dysregulation. Future directions will be to investigate other molecules important for AV that could lead to autophagic dysfunction in ET, and to determine other cargoes that may be altered due to autophagic failure implicated in ET cerebellum. Mitochondrial accumulations were observed in ET cerebellum, and the further detailed mitochondrial analysis including the levels of respiratory complex proteins and fusion/fission proteins is required to determine mitochondrial dysfunction in ET.
